# Splenic malignant fibrous histiocytoma with concurrent hypertension and epistaxis in an Alaskan malamute dog

**DOI:** 10.1186/s12917-018-1562-5

**Published:** 2018-08-13

**Authors:** Jung-Hyun Kim, Hee-Jin Kim, Sung-Jun Lee, Hun-Young Yoon

**Affiliations:** 1 Department of Veterinary Internal Medicine, Konkuk University Veterinary Medical Teaching Hospital, #120 Neungdong-ro, Gwangjin-gu, Seoul, 143-701 Korea; 2 Department of Veterinary Surgery, College of Veterinary Medicine, Konkuk University, #120 Neungdong-ro, Gwangjin-gu, Seoul, 143-701 Korea

**Keywords:** Dog, Epistaxis, Hypertension, Malignant fibrous histiocytoma

## Abstract

**Background:**

Malignant fibrous histiocytoma has been uncommonly described in dogs. Several extranasal neoplasias have been reported to result hypertensive epistaxis. There are, however, no published case reports of extranasal malignant fibrous histiocytoma with concurrent hypertension and epistaxis in dogs.

**Case presentation:**

A 10-year-old dog presented with a spontaneous massive epistaxis persisting for 5 days. The dog exhibited unstable hypertension, which was considered as a cause of epistaxis. The complete blood count, prothrombin time, and activated partial thromboplastin time were within the reference limits, and other systemic examination showed no abnormalities except for a splenic mass occupying more than one third of the abdomen. Histologic examination of the resected spleen revealed the characteristic features of a malignant fibrous histiocytoma. One week after splenectomy, the hypertension and epistaxis resolved clinically and did not recur on the 5-month follow-up.

**Conclusions:**

The dog’s blood pressure and epistaxis normalized after malignant fibrous histiocytoma resection suggesting that hypertensive epistaxis may be a rare manifestation of canine malignant fibrous histiocytoma.

## Background

Malignant fibrous histiocytoma (MFH) is a soft tissue sarcoma characterized by the presence of variably fibroblastic to less obviously histiocytic cells and variable numbers of associated non-neoplastic inflammatory cells [1, 2]. MFH most frequently affects the lung, hilar lymph nodes, mesenteric lymph nodes, liver, and spleen [3], and various reported clinical signs of MFH depend on the tumor location. MFH is associated with a rapid clinical progression, grave prognosis, and usually fatal outcome [4]. Although MFH is the most common type soft tissue sarcoma reported in humans, it has been uncommonly described in veterinary medicine [5]. A previous survey of the types of tumors occurring in dogs found that MFH comprised only 0.34% of all reported tumors [6]. Generally, MFH is reported in older dogs [2], although one report describes a case involving a puppy [7].

Epistaxis is a common and potentially severe or fatal otolaryngologic emergency in dogs that may be caused by either local disease within the nasal cavity or systemic illness [8, 9]. Canine epistaxis may be caused by various disorders, including hemostatic abnormalities, neoplasia, infection, foreign bodies, nasal parasites, hyperviscosity syndrome, and vasculitis [9]. In veterinary medicine, systemic hypertension has also been considered as a potential cause of epistaxis, although the direct mechanism remains unclear [8]. Notably, several cases of epistaxis resulting from hypertension or bleeding disorder caused by an extranasal neoplasia have been reported in human and veterinary medicine [8, 10, 11]. However, a link between extranasal MFH with concurrent hypertension and epistaxis has not been reported previously in dogs [3, 4]. In this report, we present the first description of concurrent hypertension and epistaxis in a dog with a splenic MFH, and the subsequent return of blood pressure values to within reference ranges after splenectomy. Therefore, we review the veterinary literature and suggest the possibility regarding hypertension and epistaxis in a dog with splenic MFH.

## Case presentation

### Medical history and clinical sign

A 10-year-old spayed female Alaskan malamute (body weight 38 kg) was admitted to the Konkuk University Veterinary Medical Teaching Hospital with persistent intermittent bilateral epistaxis of 5 days’ duration. Per the owner’s report, once initiated, the epistaxis did not stop for 2 h despite nasal plugging. During the initial physical examination, the dog was bright and alert, with no nasal bleeding. No purpuric spots were observed throughout the body, and an oral examination revealed no remarkable findings. Thoracic auscultation revealed no abnormal sounds in the lung and cardiac fields, and an automated oscillometric blood pressure measurement revealed mild hypertension (systolic blood pressure 148 mmHg). However, abdominal palpation revealed a large, round, firm, painful mass on the upper-middle abdomen.

### Hematology and biochemistry

Complete blood count, serum biochemistry profile, prothrombin time, and activated partial thromboplastin time analyses were performed to rule out coagulapathies, polycythemia, and thrombocytopenia as causes of epistaxis (Table 1). The complete blood count revealed neutrophilic leukocytosis (white blood cells 42.97 ×  10^9^ cells/L; reference range 6–17 × 10^9^ cells/L) and anemia (hematocrit 30%; reference range 37–55%, hemoglobin 8.7 g/dL; RI 12–18 g/dL). Serum chemistry revealed mildly elevated alkaline phosphatase activity (378 U/L; reference range 15–127 U/L) and hypoalbuminemia (2.6 g/dL; reference range 2.9–4.2 g/dL). The results of coagulation tests were within reference limits (activated partial thromboplastin time 9.6 s; reference range 14–18 s, prothrombin time 8.2 s; reference range 6.2–8.2 s).

**Table 1:** The results of coagulation tests, complete blood count, and serum biochemistry profile in a dog presenting with epistaxis

Variable	Result	Reference Interval
PT (seconds)	8.2	6.2–8.2
APTT (seconds)	9.6	14–18
WBC (× 10^9^ cells/L)	42.97	6–17
Haematocrit (%)	30	37–55
Platelet (× 10^9^ cells/L)	239	200–500
Alkaline phosphatase (U/L)	378	15–127
Total protein (g/dL)	6.8	5.4–7.4

*APTT* activated partial thromboplastin time, *PT* prothrombin time, *WBC* white blood cell

### Diagnostic imaging

Thoracic radiography revealed a mild broncho-interstitial pattern in the overall lung field and a normal cardiac size. An abdominal ultrasound examination revealed a splenic mass with a heterogeneous appearance and irregular but encapsulated borders.

**Fig. 1:**
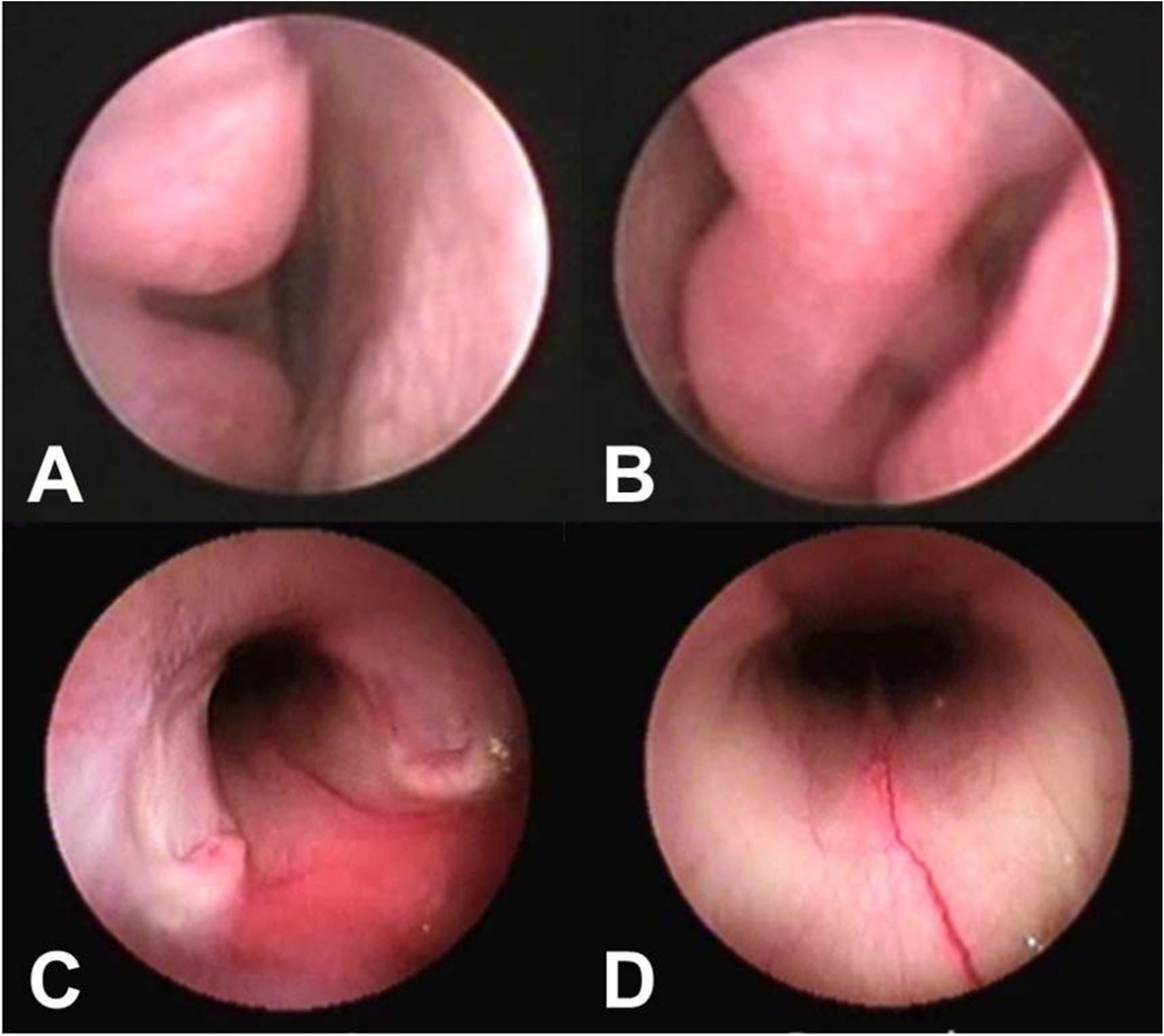
Rhinoscopy and nasopharyngoscopy did not reveal a mass occupying the nasal and nasopharyngeal regions. Mild congestion was observed in the left (**a**) and right (**b**) nasal cavities. Nasopharyngoscopy revealed congestion in the nasopharyngeal region (**c**) and engorged vessels in the nasal cavity (**d**)

**Fig. 2:**
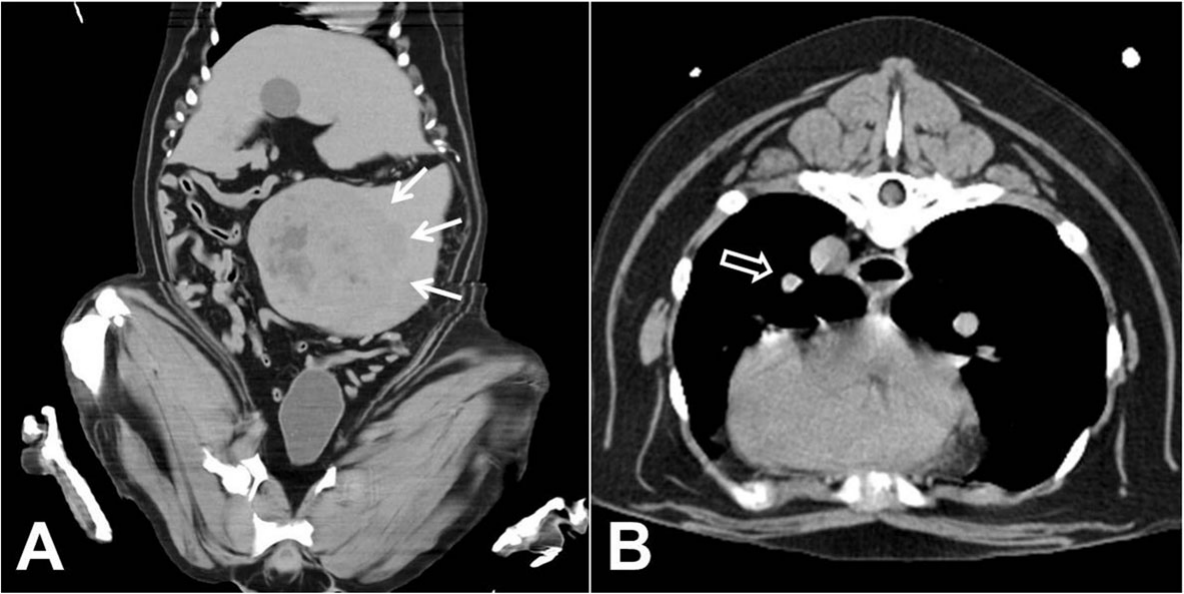
Computed tomography of the (**a**) abdomen and (**b**) thorax. **a** A mass continuous with the splenic parenchyma was detected in the mid-abdomen. Note the diffuse heterogeneous contrast enhancement of the splenic parenchyma and irregular margin (mass size: 13.2 cm × 13.5 cm × 8.4 cm) (arrows). **b** Imaging of the thorax revealed a well-circumscribed, contrast-enhanced miliary nodule on the left cranial lung lobe (open arrow)

The dog’s owner elected to pursue a rhinoscopy and computed tomography (CT) of entire body. Following anesthetization of the dog, the rhinoscopy was performed using flexible endoscopes (Fig. 1a and b). A retroflexed view of the bilateral nasal choana revealed no remarkable findings except an engorged vessel (Fig. 1c and d), and CT images revealed a normal nasal passage and intact cribriform plate. However, an abdominal CT scan detected a massive, continuous splenic mass measuring 13.2 cm × 13.5 cm × 8.4 cm in the mid-abdomen, as well as diffuse contrast-enhancement of the heterogeneous splenic parenchyma and an irregular margin (Fig. 2a). A thoracic CT scan indicated well-circumscribed, contrast-enhanced miliary nodules on the left cranial lung lobe, consistent with lung metastasis (Fig. 2b). The patient was hospitalized, during which time an episode of intractable and pulsatile epistaxis occurred without cessation for more than 1 h, despite nasal plugging. This episode caused the dog to become slightly agitated, with a temporary severe increase in blood pressure (systolic blood pressure 180–250 mmHg).

### Surgery and histopathology

**Fig. 3:**
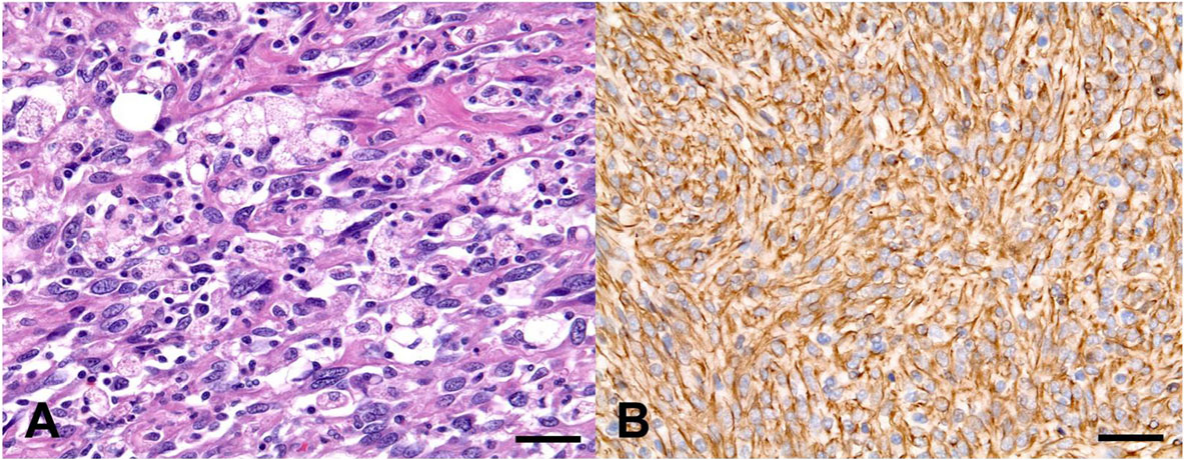
Histopathology of a malignant fibrous histiocytoma resected from the spleen. **a** Note the plump, spindle-shaped fibroblasts and histiocyte-like cells and the storiform pattern. Oval or polygonal malignant spindle cells with anisokaryosis and coarse nuclei, as well as scattered inflammatory cells, are shown (hematoxylin and eosin, magnification × 400, scale bar = 35 μm). **b** Immunohistochemically, the tumor cells were strongly positive for vimentin (magnification × 400, scale bar = 35 μm)

The owner consented to a surgical excision of the splenic tumor, and the dog underwent a surgical exploration of the abdominal cavity while in the dorsal recumbent position. A large (16 cm × 14 cm × 10 cm) mass at the tail of the spleen was found to attach to the greater omentum. The involved section of the greater omentum was resected, and a total splenectomy was performed. The abdomen was lavaged with warmed sterile saline and closed routinely. The dog recovered uneventfully with routine postoperative antibiotics. Histopathologically, the resected spleen comprised pleomorphic neoplastic cells; these included both round histiocyte-like cells and spindle cells with occasional multinucleate cells and mitotic activities ranging from one to four mitoses per high-power field (Fig. 3a). An immunohistochemistry stain for vimentin yielded a positive result (Fig. 3b), consistent with the mesenchymal origin, and the main differential diagnosis of spindle cell squamous carcinoma was excluded. A diagnosis of MFH was made based on the histologic and immunohistochemical findings.

### Treatment and outcome

Butorphanol^1^ (0.1 mg/kg intravenously every 8 h) was provided as postoperative analgesia for the first 24 h. Although intermittent minor episodes of epistaxis and transient hypertension occurred during the 7 days after splenectomy, the significant epistaxis had disappeared by the 5-month follow-up. Based on the histopathological findings, we recommended that the dog undergo adjuvant chemotherapy, but the owners refused. Five months after the first visit, however, the patient presented with acute anorexia and abdominal pain. Although her blood pressure was within normal limits (systolic blood pressure 125 mmHg), a complete blood count revealed neutrophilic leukocytosis (66.7 × 10^9^ cells/L), anemia (hematocrit 26%, hemoglobin 9.5 g/dL), and thrombocytopenia (platelets 136× 10^9^ cells/L; reference range 200–500 × 10^9^ cells/L). A serum chemistry analysis detected elevated hepatic enzyme levels (aspartate aminotransferase 84 U/L, RI 0–50 U/L, alkaline phosphatase activity 1260 U/L, gamma glutamyl transferase 14 U/L; reference range 0–7 U/L) and mild hypertriglyceridemia (173 mg/dL; reference range 10–100 mg/dL). An abdominal ultrasound revealed multiple nodules and masses of various sizes, shapes, and echogenecities that were spread diffusely throughout all liver lobes, leading to the diagnosis of metastatic liver masses and nodules. However, the owner declined further examinations and therapy because of the poor prognosis, and the patient was discharged at the owner’s request. The dog died 3 days later at home; however, the owner unfortunately refused a necropsy.

## Discussion

To the authors’ knowledge, no previous report has described concurrent hypertension and epistaxis in the dog with MFH. In the present case, the hypertension and epistaxis resolved after the surgical resection of MFH. Accordingly, this the first report of concurrent systemic hypertension and epistaxis attributable to MFH in a dog, with resolution after splenectomy. The lack of clinical reports describing these concurrent diseases processes might be attributable either to the low prevalence of splenic MFH in dogs or the fact that blood pressure evaluations are not routinely performed when abdominal masses are detected.

Epistaxis may be caused by local disease within the nasal cavity or by systemic diseases that result in hemostatic disorders [9]. In a retrospective study of 176 dogs with epistaxis, the condition was predominantly attributed to local causes (83% vs. 17% systemic) [8]. In the present case, we did not detect any mass, inflammation, infection, or foreign body in the nasal cavity and nasopharynx of the dog. However, the present dog’s indirect systolic blood pressure was above the reference range, and therefore epistaxis might be attributable to hypertension. In an earlier veterinary medicine case, excessive and tortuous vessels were observed in the caudal rhinoscopy of a hypertensive cat with epistaxis [12]. In the present case, a nasopharyngoscopic examination revealed marked vascular engorgement in the nasal mucosa. Accordingly, the resection of the splenic MFH appeared sufficient to normalize the blood pressure and resolve epistaxis in the present canine patient.

In dogs, hypertension generally develops at an older age and more commonly affects males than females [13]. The most common diseases associated with canine hypertension are renal disease, cardiac left ventricular hypertrophy, hyperadrenocorticism, pheochromocytoma, and diabetes mellitus, whereas less commonly associated diseases include primary hyperaldosteronism, hyperthyroidism, obesity, and idiopathic hypertension [14]. In the present case, unfortunately, we were unable to perform evaluations of cardiac or hormonal factors (e.g., cortisol, dopamine, aldosterone, thyroxine); however, the hypertension resolved after splenectomy. Common clinical signs of hypertension in dogs include blindness or visual disturbances, although seizures and epistaxis may also present [14]. In hypertensive veterinary patients, risk of ocular injury increases when the systolic BP exceeds 180 mmHg [14, 15]. In the present case, no ocular lesions (e.g., retinal detachment, retinal hemorrhage, retinal perivascular edema, papilledema, vitreal hemorrhage, hyphema, secondary glaucoma, retinal degeneration) were observed, possibly because the episodes of hypertension exceeding 180 mmHg were transient and returned to normal values within a week.

The surgical resection of a MFH to control concurrent systemic hypertension in a dog has not previously been reported. In the present case, the hypertension was suspected to be caused by the MFH; therefore, its removal would theoretically reduce the blood pressure to within reference limits. Indeed, after splenectomy, the systolic blood pressure in the dog returned to normal and the epistaxis subsequently disappeared. The significance of the relationship between MFH and hypertensive epistaxis in the present case is unclear because epistaxis occurs even in healthy dogs. However, given the effectiveness of MFH resection in this dog, further investigations are needed to clarify the relationship between MFH and hypertensive epistaxis.

In older dogs, splenic masses are commonly occurred and may be malignant, benign, or non-neoplastic [16]. Several studies have reported that about 2/3 of canine splenic masses are malignant, and the most prevalent malignant splenic tumor is hemangiosarcoma [16, 17]. Additionally, various sarcomas, lymphoma, and MFH have been reported as malignant splenic masses [18, 19]. A diagnosis of MFH based on histological morphology is almost insufficient [20]. Therefore, for more accurate diagnosis, additional diagnostic methods are needed, including immunology and molecular approaches. The present study report diagnosis of MFH uses not only histological morphology, but also immunohistochemistry to investigate the origin of the tumor. In both humans and dogs, vimentin expression has been widely used to confirm the mesenchymal origins of tumors [21]. In this case, hematoxylin and eosin staining and immunoreactivity for vimentin led us to conclude that the tumor was a sarcoma and to make a final diagnosis of MFH. Furthermore, three types of MFH have been reported in dogs: giant cell, inflammatory, and storiform-pleomorphic [21]. Special staining techniques assisted classification of MFH, and several studies reported both histochemical staining and immunostaining including anti-actin, anti-desmin, anti-momonocytes/macrophages antibody were able to help in the classification of MFH into the three different subgroups in dogs [21, 22]. Additional diagnostic marker such as anti-S100 has been also reported to differentiate MFH from other malignant sarcoma [23]. However, definitive immunohistochemical staining patterns have not been clearly identified for MFH in veterinary medicine [21]. As multiple morphologic types may be detected in a single tumor, MFHs are usually classified by their predominant features. Accordingly, the tumor in the dog described here is most consistent with storiform-pleomorphic type MFH based on the histopathologic examination results. In human medicine, the variants of MFH have different clinical significances, and a MFH with marked inflammation is associated with a better prognosis, compared with the storiform–pleomorphic variant [24]. By contrast, the relationship between the histopathological variant and prognosis has not been reported in the context of veterinary medicine. Generally, veterinary cases of MFH have been reported as single and often locally invasive tumors, whereas metastasis is rare [1, 6]. In the present case, evidence of metastasis to the lung was evident from a CT examination at the time of the initial diagnosis. Additionally, the dog’s condition began to deteriorate at 5 months after the initial presentation, at which time new, variously sized nodules were detected during an ultrasonographic examination of the liver.

In the present study, the nasal examinations which included CT and rhinoscopy, yielded no remarkable findings, and the cause of epistaxis remains unknown. We suggest two possibilities regarding hypertension and epistaxis in this dog with MFH. First, the dog might have previously exhibited hypertension and subsequent vascular abnormalities which is commonly referred to as a target-organ damage in the veterinary medicine [14], and would therefore have been prone to epistaxis, particularly in response to pain-induced abnormal blood pressure elevations caused by the massive splenic MFH. Second, idiopathic epistaxis might cause an arousal reaction because the nasal cavity is a region of rich autonomous innervation [25], and this anxiety might manifest as transient hypertension. Accordingly epistaxis may have also been a potent trigger for hyperresponsiveness (e.g., hypertension) in the present canine case.

## Conclusions

We report herein the clinical course of hypertensive epistaxis with the putative complication of splenic MFH in a dog. This is the first report of such a case in the literature. Although MFH is an extremely rare cause of the clinical signs observed in this dog, it should be considered in the differential diagnosis for uncontrolled epistaxis without clotting profile abnormalities. Furthermore, our findings warrant an investigation of biological behaviors in a larger number of cases and follow-up blood pressure evaluations in dogs with malignant tumors.

## Abbreviations

CT: Computed tomography
MFH: Malignant fibrous histiocytoma

## Footnotes

1 Butophan Inj, Myungmoon Pharm, Seoul, Korea.
